# Attitudes to Interpersonal Touch in the Workplace in Autistic and non-Autistic Groups

**DOI:** 10.1007/s10803-022-05710-z

**Published:** 2022-09-09

**Authors:** Tegan Penton, Natalie Bowling, Aikaterini Vafeiadou, Claudia Hammond, Geoffrey Bird, Michael J Banissy

**Affiliations:** 1 Department of Psychology, Goldsmiths, University of London, SE14 6NW London, UK; 2 School of Human Sciences, University of Greenwich, SE10 9LS London, UK; 3 School of Psychology, University of Sussex, BN1 9RH Brighton, UK; 4 MRC Social, Genetic and Developmental Psychiatry Centre, Institute of Psychiatry, Psychology and Neuroscience, King’s College London, Denmark Hill, SE5 8AF London, UK; 5 Department of Experimental Psychology, University of Oxford, OX1 3PH Oxford, UK; 6 School of Psychological Science, University of Bristol, BS8 1QU Bristol, UK

**Keywords:** Interpersonal touch, Autism, Employment, Loneliness, Wellbeing

## Abstract

Unemployment and underemployment have consistently been shown to be higher in autistic adults relative to non-autistic adults. This may be due, in part, to a lack of workplace accommodations being made for autistic people. One factor that may contribute to employment inequalities in autistic people is differences in attitudes towards interpersonal touch. This study acts as a preliminary investigation into whether employed autistic and non-autistic participants differ in their attitudes towards touch in the workplace, and in their loneliness and wellbeing. The current dataset was drawn from a larger online survey (the Touch Test) designed to explore attitudes and experiences towards touch. We found that employed autistic participants had more negative attitudes to general, social and workplace touch relative to non-autistic participants. Autistic participants also experienced greater loneliness and reduced wellbeing. Attachment-related anxiety was the only significant predictor of wellbeing in employed autistic adults. However, attachment-related anxiety, general attitudes to touch and the role of touch in the workplace predicted wellbeing in employed non-autistic adults. With regards to loneliness, general attitudes to touch and the role of touch in the workplace predicted loneliness in autistic participants. We also replicated the finding that a greater proportion of autistic participants were unemployed relative to non-autistic participants. Collectively, this research highlights the importance of considering touch in research investigating employment, and its impact on loneliness and wellbeing, in autistic participants.

Working is ubiquitous in human society. There are clear financial motivations and incentives to being employed (manifest benefits of employment) but employment can also be beneficial for one’s psychological health (Modini et al., 2016). The workplace can facilitate feelings of purpose and achievement within us, as well as provide us with a social network (latent benefits of employment, Jahoda 1982; Bryce & Haworth, 2002, 2003). Thus, there are a variety of financial, psychological, and social factors linked to employment that can impact psychological wellbeing. However, this impact may not always be positive (Stansfeld & Candy, 2006). For example, social situations in the workplace can be difficult to navigate. This can be especially challenging for those who have existing difficulties with social interaction, or for those who interact in a different way to those around them.

Autism Spectrum Disorder (hereafter ‘autism’) is a neurodevelopmental disorder characterised by social communication and interaction difficulties, and rigid and repetitive behaviours (APA, 2013). Unemployment and underemployment have consistently been shown to be higher in autistic adults relative to non-autistic adults (The National Autistic Society, 2016; Office for National Statistics, 2020; Hedley et al., 2017). Autistic people also find it more difficult to retain employment even when possessing qualities deemed valuable by employers (e.g., reliability, honesty, attention to detail; Hillier et al., 2007). This may be due, in part, to the failure of workplaces to make accommodations for autistic people (Hedley et al., 2017). However, research into other factors that may influence job retention rates, and how well autistic people adjust to workplace environments, is limited (López & Keenan, 2014). Thus, exploring factors that may contribute to employment inequalities in this group is a timely and important avenue for research.

One factor that may contribute to employment inequalities in autistic people is social challenges experienced in the workplace (Bury et al., 2021). There are many social dynamics that are important in the workplace. These can include first impressions, which are known to be important during early stages of the employment process (e.g. interviews) and when meeting colleagues for the first time (e.g. Bourdage et al., 2020; Stewart et al., 2008). They also extend to ongoing workplace interactions that can contribute to forming and maintaining relationships at work, and job satisfaction.

Several verbal and non-verbal cues can play an important role in these workplace social interactions, including facial expressions, tone of voice, and body language. For example, both verbal and non-verbal cues have been shown to contribute to differences in first impressions made by autistic people compared to non-autistic people (Sasson et al., 2017). These findings indicate that non-autistic people’s first impressions of autistic people can sometimes be more negative and contribute to a reduced likelihood of engaging in social interaction with autistic people (Morrison et al., 2019; Sasson et al., 2017).

One powerful, but often understudied, nonverbal social cue in workplace settings is the perception and experience of physical touch (Heaphy, 2017; Fuller et al., 2011). Touch is an important part of many workplace interactions. For example, social touch such as a handshake when greeting someone or touching someone on the shoulder to get their attention can be common in workplace settings (Fuller et al., 2011). Even these brief tactile interactions can impact on employment interview outcomes (Stewart et al., 2008) and impact upon wider social behaviours that can be important in the workplace (e.g., compliance, negotiating, prosocial behaviour, trait impressions, volunteering: see Gallace & Spence 2010; Saarinen, Harjunen, Jasinskaja-Lahti, Jaaskelainen, & Ravaja, 2021 for review).

Consensual, positive, interpersonal touch in the workplace has been linked to favourable impressions between staff members, including managers being perceived as more supportive, sincere, effective, and likeable (Fuller et al., 2011). Further, people can also detect and share emotions that are known to be important in the workplace through touch (e.g., gratitude, sympathy, forgiveness; App et al., 2011; Hertenstein et al., 2006; Hertenstein et al., 2009; Marler et al., 2011). Collectively, these studies highlight the potential benefits of consensual interpersonal touch in the workplace.

Whilst interpersonal touch in the workplace may be beneficial for some, there is little understanding of the social psychology of workplace touch in autistic people. This is an important gap since many autistic people experience touch differently from non-autistic people; this experience is also highly variable within the autistic community (Baranek et al., 2006; Mikkelsen et al., 2018; Thye et al., 2018). Some autistic people also show an aversion to social touch, especially when it is initiated by someone else (Kern et al., 2007). Increased sensitivity to touch has also been related to a reduced likelihood to take initiative in social interactions in autistic people (Lundqvist, 2015). Thus, the workplace may provide a particularly challenging social environment for those autistic people who experience touch differently due to an increased likelihood of interacting with, and being touched by, unfamiliar people (in contrast to a social event with friends, for example). This, in turn, may influence employment retention rates or the propensity for autistic people to seek employment.

Another factor that may influence employment prospects of autistic people is their likelihood of experiencing other co-occurring disorders. In addition to core symptoms of autism, autistic people are also highly likely to experience a range of co-occurring disorders (e.g., anxiety, depression, ADHD; Hossain et al., 2020). These can also be associated with reduced employment prospects and may manifest differently in autistic people compared to those without autism (e.g., Lerner et al., 2004; Kerns et al., 2014). Additionally, symptoms of disorders like anxiety and depression may be amplified in autistic people who feel the need to mask in given social situations (i.e. to inhibit behaviours that come more naturally and/or mimic behaviours of non-autistic people; Hull et al., 2021). These co-occurring disorders may also lead to differences in social boundaries or tolerance of social touch. For example, people who experience high levels of social anxiety preferred increased social distance between themselves and a stranger than people with lower levels of social anxiety (Perry et al., 2013). Additionally, clinical depression and symptoms of social anxiety are both associated with a dislike of social touch (Triscoli et al., 2019; Lapp & Croy, 2020). Thus, it is important to consider both core symptoms of autism, and symptoms of disorders that commonly co-occur with autism, when determining the suitability of workplace environments for autistic adults.

Other individual difference factors such as our cultural and religious backgrounds may also contribute to our experiences of and preferences for touch (Sorokowska et al., 2021). Further, it is also important to acknowledge that perceptions and experiences of touch can shift with social customs connected to touching behaviours (Saarinen et al., 2021; Simmering et al., 2013). While there is prior evidence linking consensual touch with positive outcomes in workplace settings (e.g. Fuller et al., 2011; Marler et al., 2011), several global events may have changed attitudes and experiences towards workplace touch in recent times. One example is the #MeToo movement against sexual assault and harassment. Emerging in 2017, one consequence of the #MeToo movement was to highlight the pervasiveness of non-consensual touch in the workplace. This has been connected to changes in workplace culture (e.g. Kessler et al., 2021), which may impact on people’s thoughts and experiences of touch in the workplace. There is currently a lack of research exploring how autistic and non-autistic people consider touch in the workplace in in a post-MeToo era – this study sought to fill that gap.

To do so, the current study used data from a large cross-sectional survey conducted in 2020 to investigate whether employed autistic and non-autistic participants differed in their attitudes towards interpersonal touch in the workplace, and in their loneliness and wellbeing. Second, we aimed to investigate whether differences in attitudes to touch, compared with the general population, predict loneliness and wellbeing in employed autistic people. Specifically, we sought to examine the following research questions: (1) Do employed autistic and non-autistic adults differ in their attitudes to general, social, and workplace touch? (2) Do employed autistic and non-autistic adults differ in wellbeing and loneliness? (3) Do touch-related factors predict wellbeing and loneliness in employed autistic and non-autistic participants? We also investigated whether existing findings that autistic adults are more likely to be unemployed than their non-autistic peers are replicable in our sample. Specifically, we sought to examine the hypotheses that autistic adults in our sample would have higher rates of unemployment, and lower rates of full-time employment, than non-autistic adults.

## Method

The current dataset was drawn from a large cross-sectional survey (the Touch Test) conducted in collaboration with the BBC and Wellcome Collection. This survey was predominantly designed to explore attitudes and experiences towards touch. In addition, questions related to participant demographics, mental health, and neurodiversity were included as well as measures of well-being, loneliness and personality. The study was an online self-report survey comprising several independent scales using an opportunity sample. Participants were recruited through broadcasts on the BBC and other media. There was no monetary incentive to take part. Participants were only able to take part if they were aged over 18 years old and had internet access on a computer, smart phone or tablet to participate. Data was collected between January and March 2020.

Please note that a limitation of the current study is that none of the researchers were themselves autistic. Participatory research practices have potential to improve research quality, relevance, and translational impact (Fletcher-Watson et al., 2019; Long et al., 2017).

## Pre-Registration

Methods and analyses for this project were pre-registered using the open science framework. Please see the full pre-registration available at: https://osf.io/tmnjh.

## Power

Our target sample size was a minimum of 80 participants in each group. This sample was determined using a power calculation conducted in G*Power, performed to determine the minimum number of observations required to perform a multiple linear regression with 5 predictor variables (in case we need to control for differences in reported comorbid disorders), and the minimum number of observations required to perform an ANCOVA, to detect medium effect sizes (i.e. f^2^ = 0.15, α = 0.05, β = 0.80).

## Participants

The original dataset consisted of 39,254 participant data points. A smaller number of autistic participants completed the survey compared to participants without autism. Participants self-reported whether they had Autism Spectrum Disorder due to the nature of the survey. Specifically, participants were asked if they had “any of the following disabilities, long-term conditions or impairments?” For those who selected “Autism spectrum disorder or other neurodiversity (such as dyslexia, dyscalculia)”, they were then asked “Please indicate the nature of your neurodiversity”. Participants who selected “Autism Spectrum Disorder” at this stage were included in the autistic sample. Please note, at all stages, participants were able to select multiple options.

Given the smaller number of self-reported autistic participants, a random age- and gender-matched subsample of participants without autism was selected as a non-autistic comparison group. Participants were removed if they did not complete the survey, if they lived outside of the United Kingdom, and if they did not specify their employment status. Participants were also excluded if they did not specify their age or if they did not specify their gender (male, female or non-binary). This was done to ensure that participants could be matched on these factors as other gender response types (e.g., “other” or “prefer not to say” could represent different things for different participants). Consequently, 273 autistic and 273 non-autistic individuals were included in the final sample (see Table 1 for demographic information).

**Table 1:** *Demographic information and information on co-occurring neurodiversities and conditions for autistic and non-autistic groups. SD = standard deviation. Note, groups were deliberately matched on age and gender*

		Autism	Non-autism
**Demographic Information**	**Age (mean (SD))**	47.4 (15.92) years	47.41 (15.92) years
	**Gender (Male:Female:Non-binary)**	99:155:19	99:155:19
**Co-occurring Neurodiversities**	**ADHD**	31	3
	**Dyslexia**	37	4
	**Dyspraxia**	30	0
	**Dyscalculia**	13	0
	**Tourettes**	1	0
	**Other neurodiversity**	12	1
**Other** **co-occurring Conditions**	**Hearing or speech condition**	14	6
	**Long-term health condition**	66	36
	**Mental health condition**	123	34
	**Mobility condition**	34	12
	**Visual condition**	17	6
	**Other condition**	19	17

## Materials and Variables

Below, we describe the measures relevant to the current project. For a full list of measures included in the Touch Test survey, please see the open access dataset at: https://reshare.ukdataservice.ac.uk/854471.

**Employment status.** Participants could select their employment type from 10 possible categories and could select multiple employment categories: Employed full-time, Employed occasionally, Employed part-time, Carer, Homemaker, Retired, Self-employed, Student, Unemployed, Volunteer. The number of participants that selected each category was divided by the total number of participants for each group (N = 273) to create a proportion score for each employment category for autistic and non-autistic groups.

**General attitudes to touch**. General attitudes to touch were assessed using a shortened version of the Touch Experience and Attitudes Questionnaire - TEAQ; Trotter et al., 2018). It comprises 6 subscales: Childhood Touch, Friends and Family Touch, Current Intimate Touch, Attitude to Intimate Touch, Attitude to Self-Care, and Attitude to Unfamiliar Touch. Participants used a 5-point scale to indicate whether they ‘Disagree strongly’, ‘Disagree slightly’, ’Neither agree nor disagree’, ’Agree a little’, or ‘Agree strongly’ with each statement (e.g., “I always greet my friends and family by giving them a hug.”). Questions were scored from 1 (disagree strongly) to 5 (agree strongly). Twelve items were selected from the 57-item TEAQ to be used in the current study. These items represent the 6 subscales of the questionnaire, two items from each subscale (the top two highest loading items for each subscale). Scores were summed (3 items reverse-coded) to create an overall TEAQ score (Cronbach’s Alpha in the current sample = 0.79), with higher scores indicative of more *positive* attitudes to touch.

**General attitudes to social touch.** General attitudes to social touch were assessed using the 20-item Social Touch Questionnaire – STQ; Wilhelm et al., 2001). The questions relate to 4 dimensions: giving versus receiving touch, touch involving an acquaintance versus a stranger, touch occurring in a public versus private place, or touch having sexual versus non-sexual connotations. Participants used a 5-point scale (not at all, slightly, moderately, very, extremely) to indicate how characteristic or true each statement was of them (e.g., “I feel uncomfortable when someone I don’t know very well hugs me”). Questions were scored from 0 (not at all) to 5 (extremely). Scores were summed (10 items reverse-coded) to create an overall STQ score, with higher scores indicative of more *negative* attitudes to social touch.

**Attitudes to touch in the workplace.** An ‘attitudes to touch in the workplace’ predictor was created using item 8 on the STQ (“I’d feel uncomfortable if a colleague touched me on the shoulder in public”). Scores were converted from categorical format (0–4) to numerical format (1–5). Scores for autistic participants and matched non-autistic participants were then subtracted from the mean of the wider sample of employed, non-autistic, participants (Mean = 2.061, SD = 1.163, N = 6082; note, this wider sample excludes participants selected to form the matched non-autistic sample and all participants who self-identified as autistic). This creates a difference score whereby greater differences are indicative of attitudes to touch in the workplace that deviate more from the general population-based estimate, with more negative scores indicating more negative attitudes towards touch in the workplace relative to the general population-based estimate.

**Attachment style.** Avoidant and anxious attachment styles were measured using the 12-item version of the Experiences in Close Relationships Questionnaire (ECR-12; Lafontaine et al., 2015). The anxiety items aimed to measure anxious attachment styles (characterised by someone feeling that they need others whilst also fearing abandonment and rejection; Bartholomew & Horowitz 1991). The avoidance items aimed to measure avoidant attachment styles (characterised by someone who may see others as intrusive, needy, or likely to let them down, whilst valuing their own independence and self-reliance; Bartholomew & Horowitz 1991). Participants used a 7-point scale to indicate whether they ‘Strongly disagree’, ‘Disagree’, ‘Somewhat disagree’ ‘Neither agree nor disagree’, ‘Somewhat agree’, ‘Agree’, ‘Strongly agree’ with each statement (e.g., “I worry about being abandoned.”). Questions were scored from 1 (strongly disagree) to 7 (strongly agree). Scores for the 6 anxiety items were summed (after reverse coding) to create an overall anxious attachment style score. Scores for the 6 avoidance items were summed (after reverse coding) to create an overall avoidant attachment style score. In both cases, higher scores were reflective of greater attachment-related anxiety or avoidance.

**Appropriateness of workplace touch.** A measure of perceived appropriateness of workplace touch (given by a male or female colleague or a male or female supervisor) was created. This measure was created to investigate how deviations from what the majority of non-autistic people deem as appropriate workplace touch can relate to loneliness and wellbeing in autistic and non-autistic people. Please note, we are not suggesting that non-autistic perceptions of workplace touch are ‘correct’, rather, that workplace accommodations may be built around what the majority of non-autistic people deem as appropriate and that this, in turn, may contribute to feelings of loneliness and isolation in other people (both autistic and non-autistic) who feel differently. Participants were asked to select what forms of touch (A hug, A kiss on the cheek, A handshake, A high-five, A squeeze on the arm, A pat on the shoulder, No touch at all) they felt were appropriate when given by a colleague or supervisor as a farewell gesture – participants could select multiple options. The responses were collapsed across gender and colleague type given that the primary research question does not relate to variance in scores based on these factors. Data from the wider sample of employed, non-autistic participants (excluding participants selected to form the matched non-autistic sample and all participants who self-identified as autistic) was used to determine the most frequently selected categories. Categories were then ranked based on the number of people who selected them as appropriate. Categories were ranked from most common (score of 1) to least common (score of 7). We then calculated an overall score for each autistic and non-autistic participant from the matched groups by assigning their selected categories the ranked values determined from the wider sample of employed participants (see Table 2). We then summed the ranked scores for each participant and divided this by the number of categories that participant had selected (creating an overall average score). For example, if a participant selected ‘A handshake’, ‘No touch at all’, and ‘A hug’, then we would sum the corresponding ranked values (1 + 3 + 5) and divide these by 3 (the number of categories selected), resulting in a touch appropriateness score of 3 for that participant. Lower scores reflect perceiving less touch as appropriate in the workplace. Please note, this approach differs from the preregistered analysis which was not conducted. We chose to use this approach instead of a cluster analysis as it was more appropriate given the nature of the research question. Notably, this approach allows the derivation of a single score for each participant that reflects their perceived appropriateness of touch in the workplace in comparison to the general population.

**Table 2:** *Ranking of touch gesture categories based on frequency with which they were selected as appropriate for the workplace by a wider non-autistic sample*

Ranking	Touch Gesture	Number of participants selecting category as appropriate
1	A handshake	3635
2	A pat on the shoulder	3085
3	No touch at all	2701
4	A squeeze on the arm	2601
5	A hug	2151
6	A high-five	1199
7	A kiss on the cheek	1186
	**Total sample size**	6219

**Wellbeing.** General wellbeing was measured using the Short Warwick–Edinburgh Mental Well-being Scale (Stewart-Brown et al., 2009). Participants used a 5-point scale (none of the time, rarely, some of the time, often, all of the time) to indicate the option on the scale that best described their experience of each statement over the previous 2 weeks (e.g., “I’ve been feeling optimistic about the future.”). Questions were scored from 1 (none of the time) to 5 (all of the time). Scores were summed (no items reverse-coded) to create an overall wellbeing score, with higher scores indicative of *greater* wellbeing.

**Loneliness.** Participants completed the 20-item UCLA Loneliness Scale (Russell, 1996). Based on a recent analysis of the Touch Test data examining of the 20-item UCLA and its shorter forms, we used the 4 item UCLA-LS in all analyses (Panayiotou et al., 2022; Russell et al., 1980). Item 1 (reverse coded; “I feel in tune with the people around me.”), item 13 (“No one really knows me well.”), item 15 (reverse coded; “I can find companionship when I want it.”), and item 18 (“People are around me but not with me.”) from the 20-item UCLA scale were included in the UCLA-LS. Note, that in that study the authors conclude that the 4-item UCLA-LS is a robust measure for reliably measuring loneliness across adult age groups.

Participants used a 4-point scale (Never, Rarely, Sometimes, Often) to indicate how often each of the statements was descriptive of them. Questions were scored from 1 (never) to 4 (often). Scores were summed to create an overall loneliness score, with higher scores indicative of *greater* loneliness.

## Results

### Proportion of autistic and non-autistic participants across different employment categories

A significantly higher proportion of autistic participants were unemployed relative to non-autistic participants (χ^2^(1,N = 45) = 8.74, *p* = .003, *W* = 0.44). Trends toward significant group differences were also observed for those in full-time employment (χ^2^(1, N = 162) = 3.51, *p* = .06, *W* = 0.15) and those caring for a loved one (χ^2^(1, N = 41) = 3.19, *p* = .074, *W* = 0.28), with fewer autistic participants in full-time employment and more autistic participants in caring roles compared to non-autistic participants. No group differences were observed for any other employment categories (Employed occasionally/casually, χ^2^(1, N = 31) = 0.03, *p* = .85, *W* = 0.03, Employed part-time, χ^2^(1, N = 92) = 0.21, *p* = .65, *W* = 0.05, Homemaker, χ^2^(1, N = 50) = 0.35, *p* = .55, *W* = 0.08, Retired, χ^2^(1, N = 97) = 1.02, *p* = .31, *W* = 0.1, Self-employed, χ^2^(1, N = 97) = 1.52, *p* = .22, *W* = 0.13, Student, χ^2^(1, N = 66) = 1.72, *p* = .19, *W* = 0.16, Volunteer, χ^2^(1, N = 55) = 0.51, *p* = .48, *W* = 0.1). See Table 3 for participants in each employment category.

**Table 3:** *Percentage and number of participants in each employment category*

		Participants in each employment category			
		**Autistic Group**		**Non-autistic Group**	
		**Percentage**	**Total Number**	**Percentage**	**Total Number**
**Employment Category**	**Employed full-time**	26.01	71	33.33	91
	**Employed occasionally/casually**	5.49	15	5.86	16
	**Employed part-time**	16.12	44	17.58	48
	**Home carer**	9.52	26	5.49	15
	**Homemaker**	8.42	23	9.89	27
	**Retired**	16.12	44	19.41	53
	**Self-employed**	15.75	43	19.78	54
	**Student**	13.92	38	10.26	28
	**Unemployed**	11.72	32	4.76	13
	**Volunteer**	10.99	30	9.16	25
	**Total number of participants**		273		273

## Attitudes towards touch in autistic and non-autistic people in employment

103 autistic participants and 103 non-autistic participants were included in the final sample (See Table 4). Please note, this sample size is smaller than that in the frequency analysis because participants were removed if they stated that they were “self-employed” or “retired” in addition to other removal criteria (see Participants subsection in Methods). We first applied the exclusion criteria to the autistic sample, and then selected a subsample of age- and gender-matched non-autistic participants.

**Table 4:** *Demographic information for subsample of autistic individuals in employment and a matched group of employed non-autistic individuals. SD = standard deviation*

	Autism	Non-autism
**Age (mean (SD))**	43.16 (14.22) years	43.17 (14.22) years
**Gender (Male:Female:Non-binary)**	38:62:3	38:62:3

**Fig. 1:**
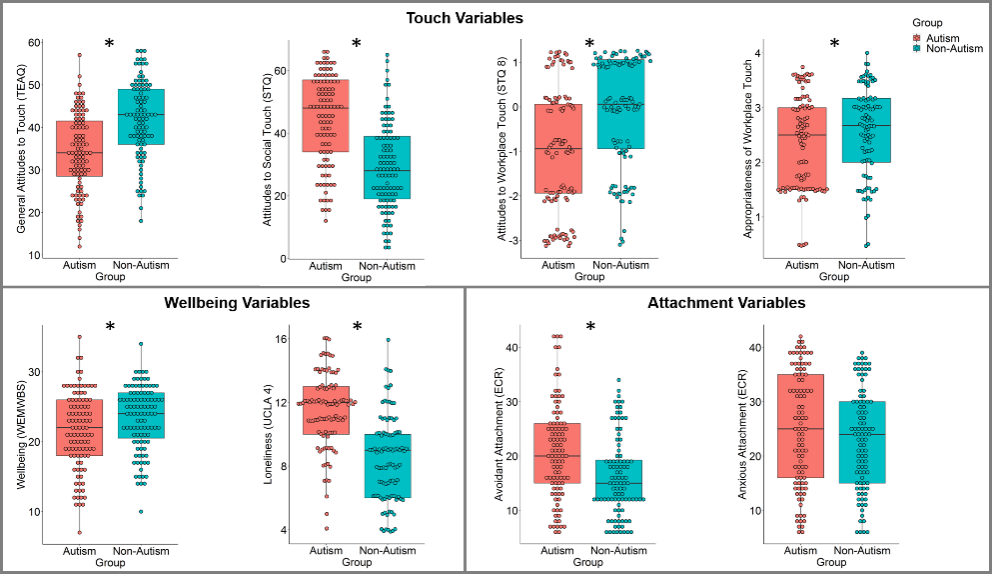
Group differences in touch variables (top panel), wellbeing variables (lower left panel), and attachment variables (lower right panel)

**Group comparisons.** Independent t-tests were conducted to compare autistic and non-autistic groups on variables of interest. A summary of descriptive statistics can be seen in Fig. 1. Autistic participants had significantly more negative general attitudes to touch and significantly more negative attitudes to social touch (general touch: [*t*(193) = -5.805, *p* < .001, d = 0.83], social touch: [*t*(203) = 7.657, *p* < .001, d = 1.07]). They also deviated further from the general population estimate of attitudes to touch in the workplace, displaying more negative attitudes towards touch in the workplace relative to the non-autistic matched sample (*t*(203) = -4.657, *p* < .001, d = 0.65). A one-sample t-test also confirmed that autistic attitudes to workplace touch were significantly more negative than those of the general population (*t*(102) = -7.182, *p* < .001, d = 0.71). Attitudes to workplace touch in the non-autistic matched sample did not significantly differ from the general population (*t*(101) = -1.09, *p* = .278, d = 0.11). Relatedly, autistic participants perceived workplace touch as less appropriate than non-autistic participants (*t*(193) = -1.978, *p* = .049, d = 0.29). In addition to touch-related variables, autistic participants also reported reduced wellbeing and greater loneliness relative to non-autistic participants (wellbeing: [*t*(204) = -2.669, *p* = .008, d = 0.37], loneliness: [*t*(193) = 8.332, *p* < .001, d = 1.19]).

Autistic participants also reported greater attachment-related avoidance, but not anxiety, relative to non-autistic participants (avoidance: [*t*(192) = 1.382, *p* < .001, d = 0.57], anxiety: [*t*(1.384) = 192, *p* = .168, d = 0.2]).

## Predictors of loneliness and wellbeing in employed autistic individuals

Hierarchical regression analysis was used to identify predictors of loneliness and wellbeing in employed autistic individuals. In particular, hierarchical regressions were used to determine whether attitudes to touch in the workplace predicted loneliness and wellbeing after accounting for variance due to ‘control’ variables (age, general attitudes to touch, attachment style, and response to the question “Do you think touch plays an important role in your work”). Control variables were entered in to the first step of the regression and attitudes to touch in the workplace was then added at the second step. Regressions were performed separately for loneliness and wellbeing dependent variables. Standardised betas are reported.

**General wellbeing.** Attachment-related anxiety was the only significant predictor of general wellbeing in the first step of the model (*b* = − 0.407, *t* = -4.459, *p* < .001) with greater attachment-related anxiety predictive of reduced wellbeing in employed autistic participants. The overall model was significant (F(5,92) = 6.85, R^2^ = 0.271, p < .001). The model remained significant when adding attitudes to workplace touch into the model, but this did not significantly increase the amount of variance explained by the model (R^2^ Change < 0.001, p = .875). Attachment-related anxiety remained a significant predictor of wellbeing (*b* = − 0.407, *t* = -4.427, *p* < .001). As above, no other variables were significant predictors of wellbeing (age: [*b* = 0.163, *t* = 1.725, *p* = .088], general attitudes to touch: [*b* = 0.194, *t* = 1.548, *p* = .125], attachment-related avoidance: [*b* = − 0.186, *t* = -1.694, *p* = .094], touch role in the workplace: [*b* = 0.083, *t* = 0.795, *p* = .429], attitudes to workplace touch: [*b* = 0.016, *t* = 0.158, *p* = .875]).

**Loneliness.** All variables entered in the first step of the model were significant predictors of loneliness in employed autistic people except for age (general attitudes to touch: [*b* = − 0.263, *t* = -2.356, *p* = .021], attachment-related avoidance: [*b* = 0.307, *t* = 3.111, *p* = .002], attachment-related anxiety: [*b* = 0.175, *t* = 2.11, *p* = .038], touch role in the workplace: [*b* = − 0.234, *t* = -2.519, *p* = .014]; age: [*b* = 0.165, *t* = 1.951, *p* = .054]). Specifically, more negative attitudes to touch and greater attachment-related avoidance and anxiety were all predictive of greater loneliness, whilst more positive general attitudes to touch and a greater role of touch in the workplace were predictive of reduced loneliness. The overall model was significant (F(5,92) = 12.076, R^2^ = 0.396, p < .001). The addition of attitudes to workplace touch did not significantly increase the amount of variance explained (R^2^ Change < 0.001, p = .928) and Attitudes to workplace touch was not a significant predictor of loneliness in the full model (*b* = − 0.008, *t* = − 0.09, *p* = .928), and nor was age (*b* = 0.166, *t* = 1.934, *p* = .056). All other predictors remained significant (general attitudes to touch: [*b* = − 0.261, *t* = -2.287, *p* = .025], attachment-related avoidance: [*b* = 0.305, *t* = 3.052, *p* = .003], attachment-related anxiety: [*b* = 0.175, *t* = 2.095, *p* = .039], touch role in the workplace: [*b* = − 0.232, *t* = -2.436, *p* = .017]).

## Predictors of loneliness and wellbeing in employed non-autistic people

Please note, the analyses in this subsection were not pre-registered. We have chosen to include them as it allows the reader to compare predictors of loneliness and wellbeing in employed autistic and non-autistic participants. Variables were entered into models in the same order as above.

**General wellbeing.** General attitudes to touch, Attachment-related anxiety and the role of touch in the workplace were all significant predictors of general wellbeing in the first step of the model (general attitudes to touch: [*b* = 0.277, *t* = 2.821, *p* = .006], attachment-related anxiety: [*b* = − 0.345, *t* = -3.843, *p* < .001], touch role in the workplace: [*b* = 0.208, *t* = 2.19, *p* = .031]). More positive general attitudes to touch and a greater role of touch in the workplace were predictive of greater wellbeing whilst greater attachment-related anxiety was predictive of poorer wellbeing. The overall model was significant (F(5,89) = 10.361, R^2^ = 0.368, p < .001). The model remained significant when adding Attitudes to workplace touch into the model (F(6,88) = 8.608, R^2^ = 0.37, p < .001), but this addition did not significantly increase the amount of variance explained(R^2^ Change = 0.002, p = .605). General attitudes to touch, Attachment-related anxiety and the role of touch in the workplace remained significant predictors of general wellbeing in the second step of the model (general attitudes to touch: [*b* = 0.3, *t* = 2.767, *p* = .007], attachment-related anxiety: [*b* = − 0.348, *t* = -3.851, *p* < .001], touch role in the workplace: [*b* = 0.219, *t* = 2.42, *p* = .027]). However, Age, Attachment-related avoidance, and Attitudes to workplace touch were not significant predictors of wellbeing (age: [*b* = 0.062, *t* = 0.667, *p* = .506], attachment-related avoidance: [*b* = − 0.026, *t* = -0.277, *p* = .783], attitudes to workplace touch: [*b* = − 0.052, *t* = -0.519, *p* = .605]).

**Loneliness.** General attitudes to touch, Attachment-related anxiety and Attachment-related avoidance were all significant predictors of loneliness in the first step of the model (general attitudes to touch: [*b* = − 0.371, *t* = -3.861, *p* < .001], attachment-related anxiety: [*b* = 0.238, *t* = 2.706, *p* = .008], attachment-related avoidance: [*b* = 0.187, *t* = 2.082, *p* = .040]). More positive general attitudes to touch were predictive of reduced loneliness whilst greater attachment-related anxiety and avoidance were predictive of greater loneliness. The overall model was significant (F(5,89) = 11.561, R^2^ = 0.394, p < .001). The model remained significant when adding Attitudes to workplace touch into the model, but this did not significantly influence variance explained (F(6,88) = 9.677, R^2^ = 0.398, p < .001; R^2^ Change = 0.004, p = .460). General attitudes to touch and Attachment-related anxiety remained significant predictors of loneliness in the second step of the model (general attitudes to touch: [*b* = − 0.404, *t* = -3.807, *p* < .001], attachment-related anxiety: [*b* = 0.242, *t* = 2.737, *p* = .008]). However, Age, Attachment-related avoidance, Touch role in the workplace, and Attitudes to workplace touch were not significant predictors of loneliness in the second step of the model (age: [*b* = − 0.006, *t* = -0.071, *p* = .943], attachment-related avoidance: [*b* = 0.174, *t* = 1.891, *p* = .062], touch role in the workplace: [*b* = − 0.137, *t* = -1.434, *p* = .155], attitudes to workplace touch: [*b* = 0.073, *t* = 0.742, *p* = .460]).

## Comparison of Predictors of loneliness and wellbeing in employed autistic and non-autistic individuals

In order to investigate whether touch-related factors differentially predicted wellbeing and loneliness in autistic and non-autistic participants, we ran an additional regression for each of the two dependent variables (DVs) including the interaction terms for each of the independent variables (IVs; age, general attitudes to touch, attachment style, response to the question “Do you think touch plays an important role in your work”, and attitudes to touch in the workplace) multiplied by group (autistic or non-autistic participants). To do this, we first recoded the group variable such that autistic participants were assigned a value of 1 and non-autistic participants were assigned a value of -1. We then mean-centred the scores of the other independent variables by subtracting the mean for the IV from each participant’s score. We then multiplied these mean-centred scores by the group value (1 or -1). These new scores were then entered into a regression model with the original IVs and group. Please note, this analysis deviates from the pre-registered analysis. This was to allow for the inclusion of the interaction terms.

**General wellbeing.** The overall model was significant (F(13,179) = 6.967, R^2^ = 0.336, p < .001), and age, General attitudes to touch and Attachment-related anxiety were all significant predictors of general wellbeing (see Table 5). Older age and more positive general attitudes to touch were predictive of greater wellbeing whilst greater attachment-related anxiety was predictive of poorer wellbeing. No other variables significantly predicted wellbeing (see Table 5). Importantly, we did not find an effect of group in the current analysis suggesting that touch-related factors did not differentially predict wellbeing in autistic and non-autistic participants.

**Table 5:** *Standardised beta values, t-statistics, and p-values for each of the predictor variables in the model. Significant predictors (p < .05) appear in bold and are marked with an asterisk*

	Standardised Beta Value	t-statistic	p-value
**Age**	0.134	1.981	0.049*
**Attitudes to touch (TEAQ)**	0.246	2.588	0.010*
**Avoidant attachment (ECR)**	− 0.137	-1.781	0.077
**Anxious attachment (ECR)**	− 0.385	-5.959	< 0.001*
**Role of touch in work**	0.124	1.625	0.106
**Attitudes to workplace touch (STQ item 8)**	− 0.006	− 0.075	0.940
**Group**	− 0.008	− 0.117	0.907
Age * Group	0.063	0.917	0.361
Attitudes to touch * Group	− 0.032	− 0.371	0.711
Avoidant attachment * Group	− 0.078	-1.102	0.272
Anxious Attachment * Group	− 0.042	− 0.650	0.517
Role of touch in work * Group	− 0.050	− 0.663	0.508
Attitudes to workplace touch * Group	0.033	0.454	0.651

**Loneliness.** Loneliness. The overall model was significant (F(13,179) = 18.073, R2 = 0.568, p < .001), and General attitudes to touch, and Attachment-related avoidance and anxiety, the role of touch in the workplace, and group were all significant predictors of loneliness (see Table 6). More positive general attitudes to touch and a greater touch role in the workplace were predictive of reduced loneliness whilst greater attachment-related anxiety and avoidance were predictive of greater loneliness. Autistic participants also reported feeling more lonely than non-autistic participants. No other variables significantly predicted loneliness (see Table 6).

**Table 6:** *Standardised beta values, t-statistics, and p-values for each of the predictor variables in the model. Significant predictors (p < .05) appear in bold and are marked with an asterisk*

	Standardised Beta Value	t-statistic	p-value
**Age**	0.087	1.600	0.111
**Attitudes to touch (TEAQ)**	− 0.283	-3.687	< 0.001*
**Avoidant attachment (ECR)**	0.217	3.510	0.001*
**Anxious attachment (ECR)**	0.166	3.191	0.002*
**Role of touch in work**	− 0.169	-2.747	0.007*
**Attitudes to workplace touch (STQ item 8)**	0.019	0.325	0.745
**Group**	0.331	5.949	< 0.001*
Age * Group	0.073	1.323	0.188
Attitudes to touch * Group	0.080	1.164	0.246
Avoidant attachment * Group	0.028	0.492	0.623
Anxious Attachment * Group	− 0.048	− 0.920	0.359
Role of touch in work * Group	− 0.041	− 0.666	0.506
Attitudes to workplace touch * Group	− 0.038	− 0.650	0.516

## Discussion

In the current study we aimed to investigate differences in employment status between autistic and non-autistic groups. In addition, we aimed to investigate differences in attitudes to interpersonal touch, and their value in predicting loneliness and wellbeing, in autistic and non-autistic employed adults. We also investigated autistic and non-autistic group differences in factors related to touch in the workplace and wellbeing and loneliness. Finally, we investigated predictors of individual differences in wellbeing and loneliness in employed autistic and non-autistic participants. We discuss these in turn below.

## Group differences in employment status

We found that a significantly greater proportion of autistic participants were unemployed relative to non-autistic participants. This is in line with previous research showing higher levels of unemployment in autistic adults relative to non-autistic adults (The National Autistic Society, 2016; Office for National Statistics, 2020; Hedley et al., 2017). We did not find any other significant differences between the two groups in other categories of employment status. This may appear inconsistent with previous research that typically also demonstrates a smaller proportion of autistic adults in employment (The National Autistic Society, 2016; Office for National Statistics, 2020; Hedley et al., 2017). However, this may be due to the fact that our non-autistic sample also included people with physical disabilities and mental health problems. Whilst not significant, we did observe a trend in the same direction as previous research where a smaller proportion of autistic adults were in full-time employment relative to non-autistic adults.

## Group differences in factors related to touch in the workplace

Employed autistic participants had significantly more negative attitudes to both general and social touch. They also deviated further from the non-autistic, general population estimate of the appropriateness of various types of touch in the workplace. Specifically, the autistic group displayed more negative attitudes towards touch in the workplace relative to both the non-autistic matched sample, and the general population estimate. Autistic participants also perceived touch in the workplace as less appropriate than matched non-autistic participants. Collectively, these findings highlight stark differences between attitudes to touch, and related factors, in employed autistic and non-autistic people.

This is important when considering how people are perceived when they engage or do not engage in touch in the workplace. Even if accommodations are made to avoid touch in an interview or workplace setting for people who prefer not to touch, avoiding touch may still hinder employment prospects or building of workplace relationships. Prior work has shown that engaging in touch in social interactions can lead to more positive outcomes and evaluations (e.g., Gallace & Spence 2010; Saarinen et al., 2021 for review); that touch may be a useful tool to help build relationships in a workplace setting (Heaphy, 2017), including increased positive evaluations of supervisors in the workplace (Fuller et al., 2011; but see Fuller et al., 2017); and that touch can impact on behaviours that are relevant to workplace outcomes (e.g. job interview outcomes, negotiations, perceptions of emotional sincerity; Marler et al., 2011; Schroeder et al., 2019; Stewart et al., 2008). Therefore, more negative attitudes to general, social, and workplace touch in autistic people may have a very real impact on outcomes of social interaction in workplace settings.

There is also a possibility that autistic people may engage in touch-related behaviours despite being uncomfortable doing so. This type of behaviour could be engaged in an effort to camouflage or mask one’s own preferences to better ‘fit-in’ with their non-autistic colleagues. Masking behaviours are often associated with increased social exhaustion and mental health difficulties in autistic people (Hull et al., 2017, 2021). Therefore, by better understanding differences in how autistic people view and engage with touch in the workplace, it may be possible for employers to build greater awareness, and workplace norms, around the nuanced nature of touch in everyday workplace interactions (e.g., engaging in handshakes and implications of this; see Schroeder et al., 2019; Stewart et al., 2008). Doing so may support improvements in making workplace environments more inclusive for the needs of all employees.

**Individual differences in wellbeing in employed autistic people.** Attachment-related anxiety was the only significant predictor of general wellbeing in our group of employed autistic adults, whereby greater attachment-related anxiety predicted reduced wellbeing. Similarly, Attachment-related anxiety, along with general attitudes to touch and the role of touch in the workplace were all significant predictors of general wellbeing in non-autistic employed participants (more positive attitudes to touch and a greater touch role in the workplace predicted greater wellbeing whilst higher attachment-related anxiety predicted poorer wellbeing). The relationship between attachment-related anxiety and wellbeing is consistent with previous literature (Bekker & Croon, 2010; Davis et al., 2016) and observed in both groups in the current study. This may suggest that mechanisms which contribute to the relationship between attachment-related anxiety and wellbeing are similar in autistic and non-autistic participants. The fact that general attitudes to touch, and the role of touch in the workplace, only significantly predicted wellbeing in non-autistic participants may suggest that touch-related factors impact wellbeing to a greater degree in non-autistic participants. However, our regression analysis including both groups did not find evidence to support the hypothesis that touch-related factors impact wellbeing differently in autistic and non-autistic participants.

In line with the finding that touch-related factors impact wellbeing in non-autistic but not autistic participants, previous research has shown that some autistic participants experience touch differently and so may find it less rewarding. Research shows that affective touch activates c-afferent fibres (related to ‘liking’ of touch). Some autistic people show differences in the amount and functioning of c-afferent fibres relative to non-autistic participants. Thus, touch may be less rewarding or likeable for some autistic participants, and may therefore not relate to increased general wellbeing to the same degree as in non-autistic participants or may be more variable in autistic individuals (Kaiser et al.,^2016^; Riquelme et al., 2016; Silva & Schalock, 2016; see also Thye et al., 2018, for review).

**Individual differences in loneliness in employed autistic people.** All variables entered into the model were significant predictors of loneliness in employed autistic people except for age and attitudes to workplace touch. More negative attitudes to touch, greater attachment-related avoidance and greater attachment-related anxiety all predicted greater loneliness. A greater role of touch in the workplace predicted reduced loneliness in employed autistic people. In employed non-autistic people, general attitudes to touch, attachment-related anxiety and attachment-related avoidance (only in the first step of the model) were significant predictors of loneliness. More positive general attitudes to touch were predictive of reduced loneliness whilst greater attachment-related anxiety and avoidance were predictive of greater loneliness.

Touch is ubiquitous in human society and so more negative attitudes to touch may increase feelings of social exclusion and loneliness. Research shows that engaging in social touch can aid in the formation of interpersonal relationships, and engaging in touch at a young age predicts social skills in later life (see Cascio et al., 2019, for review). Differing responses to touch also correlate with social (and non-social) symptoms in autism (Foss-Feig et al., 2012). As a consequence, negative attitudes to touch may hinder one’s social development and this, in turn, may increase feelings of loneliness. Additionally, more negative attitudes to touch or increased sensitivity to touch may reduce the likelihood of social touch occurring or may increase the likelihood of refusing someone else’s touch gesture (avoiding a hug, for example; Lundqvist 2015). Both of these scenarios could affect how likely one is to form social relationships and social bonds (for review see Dunbar 2010), which may help to explain why attitudes to touch predict loneliness in autistic and non-autistic participants.

The role of touch in the workplace did not predict wellbeing of autistic participants, but it did predict loneliness. This relationship was not observed in our non-autistic participants, but may point to the benefits of considering the role of touch in the workplace in autistic participants who enjoy this element of social interaction. It is important to note that this relationship may also simply reflect that autistic participants who like touch are more likely to work in jobs that require a greater touch role. Therefore, further research is needed to determine the underlying factors contributing to this effect.

**Limitations & Conclusions.** Due to the nature of the survey used to collect the data, we were not able to verify self-reported diagnoses of autism. Thus, it is possible that some people without a formal diagnosis identified as autistic. It may also be that our measures of workplace touch were not sensitive enough to detect smaller individual differences given that these were based on responses to single items on questionnaires. This study acts as a preliminary investigation of attitudes towards touch in the workplace in autistic people. We show that employed autistic participants have more negative attitudes to general, social and workplace touch relative to non-autistic participants. Moreover, general attitudes to touch and the role of touch in the workplace predicted loneliness in autistic participants. However, measures of attitudes to workplace touch did not significantly predict loneliness or wellbeing in autistic participants. Thus, our work highlights the importance of considering touch and its impact on loneliness and wellbeing in autistic participants, but suggests that general attitudes to touch, rather than attitudes towards workplace touch specifically, and their relationship with loneliness should be further explored in autism. It may also be the case that differences in other forms of non-verbal communication (e.g. gestures, body language) better predict loneliness and wellbeing in employed autistic people. Collectively, this highlights the importance of investigating the role of touch, alongside other verbal and non-verbal cues, in workplace interactions in autistic people.
